# Improving Hospital Efficiency and Cost Management: A Systematic Review and Meta-Analysis

**DOI:** 10.7759/cureus.71721

**Published:** 2024-10-17

**Authors:** Sultan A Almehwari, Ibrahim S Almalki, Bassam A Abumilha, Basim H Altharwi

**Affiliations:** 1 Family and Community Medicine, King Fahad Armed Forces Hospital, Jeddah, SAU; 2 Family Medicine, Prince Sultan Hospital, Al Madinah, SAU

**Keywords:** cost-effectiveness, healthcare, hospital efficiency, quality of care, reduction of healthcare costs, review article

## Abstract

Internationally, improving hospital efficiency and reducing costs in the healthcare sector is a goal that requires major effort and successful collaboration. Most hospitals have a dedicated department responsible for quality control, and this is where many of the challenges to efficiency and cost reductions arise. The purpose of this study is to review the literature on the successful improvement of hospital efficiency and cost reduction with no negative impact on the quality of patient care.

For this study, we conducted a systematic review and meta-analysis. We screened the available data from 2014 to 2024 in various respected databases, including the Cumulated Index to Nursing and Allied Health Literature (CINAHL) (via EBSCO), the Cochrane Library (via Wiley), PubMed, Google Scholar, and Scopus. The selected articles (n = 7) met the criteria set by the Preferred Reporting Items for Systematic Reviews and Meta-Analyses (PRISMA) guidelines, ensuring their quality and relevance. The study designs that were included are randomized clinical trials, systematic literature reviews, and prospective and retrospective cohort studies. The selected studies represent a wide range of programs and approaches that were adopted to address the issue of hospital efficiency and cost reduction, for example, the Plan-Do-Study-Act problem-solving model and a telemedicine program. These approaches achieved a 25%-50% reduction in costs, allowing for the reallocation of resources and, ultimately, an improvement in the quality of care (QoC). Regarding hospital efficiency, hospitals were encouraged to explore systems to support patient care that did not simply involve new equipment and would help reduce the supply shortage.

The findings of our study provide valuable insights that can act as a foundation for policymakers tasked with improving hospital efficiency and cost-effectiveness. On the one hand, this findings emphasize the importance of focusing on the provision of quality service, encouraging collaboration, and creating tailored solutions. On the other hand, this focus will help hospitals achieve systems that ensure efficiency, promote sustainable outcomes, and improve cost management in healthcare.

## Introduction and background

Healthcare systems worldwide face challenges that stem from the need to contain costs and improve the quality of care (QoC), with hospitals playing a crucial role in this context [1]. Studies have found that hospitals often encounter long-term financial deficits and the risk of bankruptcy while striving to meet QoC objectives at the national and international levels. The association between a healthcare provider’s financial performance and QoC has been a subject of research interest, with evidence suggesting a two-way association: Financially stable providers may offer better QoC due to their capacity to invest in new technology and attract skilled staff. In turn, better QoC may lead to financial gains [1].

This study provides a comprehensive overview of the research, outlining the background, aim, and scope of each selected study. This study also highlights the significance of the research in the context of hospital efficiency and cost management. This is particularly important given the identified barriers to efficiency improvement, such as continuous restructuring, delays in performance reporting, and the prioritization of cost reduction over service enhancement [2].

Moreover, this study emphasizes the need to measure the financial and operational efficiency of hospitals, identifying factors influencing financial efficiency and proposing improvement strategies. These insights underscore the significance of the research in addressing critical issues related to hospital efficiency and cost management, thereby contributing to the advancement of healthcare management practices.

The goal of this study is to identify the crucial components that would constitute a clear roadmap for improving hospital efficiency and reducing costs. By conducting a systematic review and meta-analysis, we aim to evaluate the effectiveness of measures suggested in the literature by exploring their impact on various aspects of hospital operations, including resource utilization, financial performance, and QoC.

## Review

Method

The methodology employed in this study follows the Preferred Reporting Items for Systematic Reviews and Meta-Analysis (PRISMA) guidelines for systematic reviews, ensuring a comprehensive and transparent approach to the research process [3].

Search Strategy

The rigorous approach to study selection and inclusion criteria demonstrates this author’s commitment to uphold the standards for systematic reviews and meta-analyses outlined by the PRISMA guidelines. This methodology not only provides a clear understanding of the systematic and analytical frameworks utilized but also ensures the reliability and validity of the findings presented in the study [4]. The defined scope of the study encompasses an in-depth investigation into the database sources, which include the Cumulated Index to Nursing and Allied Health Literature (CINAHL) (via EBSCO), the Cochrane Library (via Wiley), PubMed, Google Scholar, and the Scopus database.

*Selection and Data Collection Process * 

**Figure 1:**
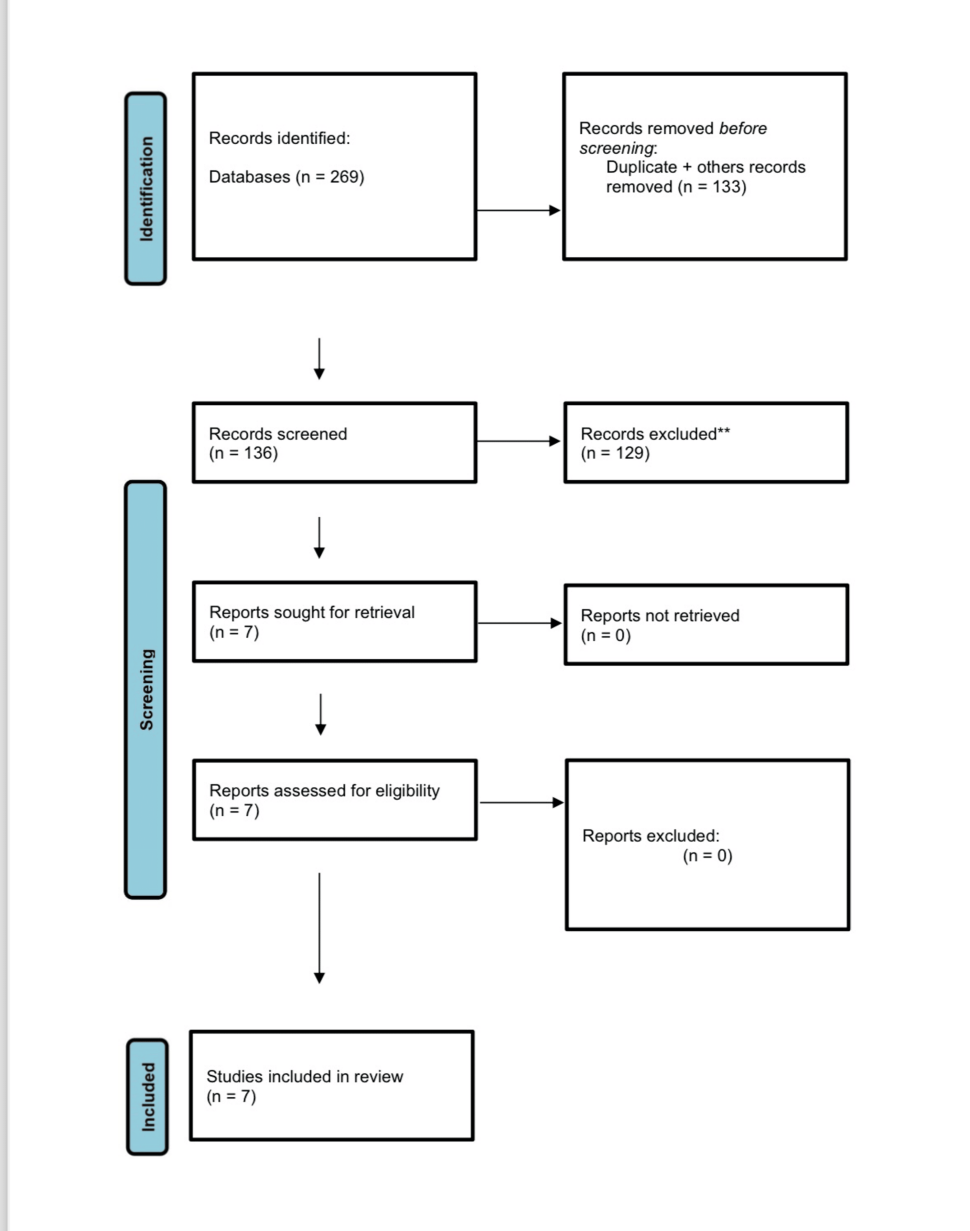
PRISMA flow chart PRISMA: Preferred Reporting Items for Systematic Reviews and Meta-Analysis

Data collection and analysis followed a systematic approach in line with PRISMA guidelines. We screened the available data in the definitive databases listed above from 2014 to 2024. Initially, a total of 269 studies were retrieved from the various databases. After the removal of duplicates, 136 unique studies remained. After screening the titles and abstracts, 29 studies were excluded. The reasons for exclusion, presented in Figure 1, include "descriptive or review," "not in healthcare facility," and "other type of study" [5,6].

After a thorough evaluation, (n = 7) studies were included in the final review. This step ensured that the selected studies met the criteria of quality and relevance [7-13]. The designs of the selected studies are randomized clinical trials, systematic literature reviews, and prospective and retrospective cohort studies.

Data Extraction and Heterogeneity

Data extraction was preformed independently by two reviewers using a standardized data extraction form to minimize bias and ensure consistency. Extracted data included study characteristics, setting or department, conclusion of the study, and recommendation. In case of discrepancies, a third reviewer was consulted for resolution.

To assess heterogeneity among the studies, we used the I2 statistic, which quantifies the percentage of total variation across studies due to heterogeneity rather than chance. A threshold of I2 > 50% indicated substantial heterogeneity, prompting further exploration of potential sources.

Meta-Analysis Techniques

For data analysis and meta-analysis, we utilized RevMan software (The Cochrane Collaboration), which facilitated the synthesis of results and the assessment of statistical significance. Meta-analysis is a systematic process that involves integrating and analyzing the results of multiple studies to achieve evidence synthesis. In addition, a meta-analysis pools the results of various studies to obtain a summary estimate. The process involves framing a research question, conducting a comprehensive literature search, assessing the quality of the included studies, evaluating heterogeneity, estimating the summary effect size using fixed and random effects models, assessing publication bias, and utilizing the population, intervention, comparison, and outcome (PICO) framework for assimilating relevant studies. Usually, meta-analyses are conducted for randomized controlled trials, quasi-experimental studies, diagnostic and screening studies, and observational epidemiological studies involving cohort and case-control studies [14,15].

Results

Among the (n = 7) studies included, most of the efficiency models adopted by hospitals were generated by the hospital’s department of quality control. In fact, this department played an important role in generating protocols to improve efficiency. However, two major challenges facing the department of quality control are resistance to change and the ongoing demands of running a hospital effectively. Both challenges have the potential to impact on patient care. It is therefore important to create a protocol for the continuous evaluation of measures to improve efficiency and reduce costs. Putting such protocols in place could help with the development of new strategies to reduce costs and improve hospital efficiency while maintaining QoC.

Table 1 presents the cost reduction and improved efficiency effects that were reported in the selected articles. These observations can help shape the formulation of a new pathway, or act as a guideline, for the re-allocation of resources and observation of demand while optimal QoC is provided in fulfilment of the healthcare institution’s goals.

**Table 1:** Descriptive characteristics of included studies EULAR: The European Alliance of Associations for Rheumatology; PUG: percutaneous ultrasound gastrostomy; LOS; length of stay; IBD: inflammatory bowel diseases; PD: Project Dulce; PD-TE: Project Dulce with technology enhancement; HIE: Health Information Exchange

Reference	Author	Country, year of publication	Type of study	Setting/department	Conclusion	Recommendation
7	Xing et al.	China, 2024	Prospective cohort study	Department of Pharmacy	Drug cost decreased by 20.82%	Employ Plan-Do-Study-Act methodology to establish a multidimensional pharmaceutical intervention
8	Bernard et al.	France, 2022	Randomized controlled trial	Clinical Research and Epidemiology Unit	97.8% chance of being cost effective	Implementing EULAR* recommendations, more efficient and less expensive than conventional care
9	Marshall et al.	USA, 2022	Retrospective cohort study	Department of Pulmonary and Critical Care Medicine	Cost reduction per patient savings of US$26,621	Bedside PUG* leads to decreased LOS* and total hospital costs
10	De Jong et al.	Maastricht, 2020	Randomized clinical trial	Department of Internal Medicine	Telemedicine resulted in lower mean annual costs of €547/patient [US$612]	Telemedicine with IBD* coach is cost saving and has a high probability of being cost effective for patients with IBD
11	Gilmer et al.	USA, 2019	Randomized clinical trial	University of California	Intervention costs were US$1448 for PD* and US$1740 for PD-TE*, interventions were cost-effective under time horizons of 15 to 20 years	Implementation of PD-TE is warranted
12	Sadoughi et al.	Iran, 2018	Systematic literature review	Health Management and Economics Research Center	15 of HIE* studies (60%) demonstrated positive financial effects	Findings suggest positive financial and quality impact related to HIE use in healthcare. However, further research is needed
13	Frederix et al.	Belgium, 2016	Multicenter randomized controlled trial	Department of Cardiology	The total average cost per patient was significantly lower in the (telerehabilitation) intervention group (€2156 ± €126) than in the control group (€2720 ± €276) (p = 0.01)	Healthcare resources should best be allocated in the era of exploding need

Most of the studies selected for this review agreed on important principles that, if applied, would create change and help hospitals achieve their goals. The principles of Plan-Do-Study-Act (PDSA) and telemedicine brought about major change, as demonstrated in improved hospital efficiency and reduced costs. These approaches showed a reduction of cost of around 25%-50%. This saving meant that resources could be re-allocated, and institutions were provided with alternative investment options. Moreover, optimal QoC was maintained, which is the overarching goal of all staff at these institutions.

**Figure 2:**
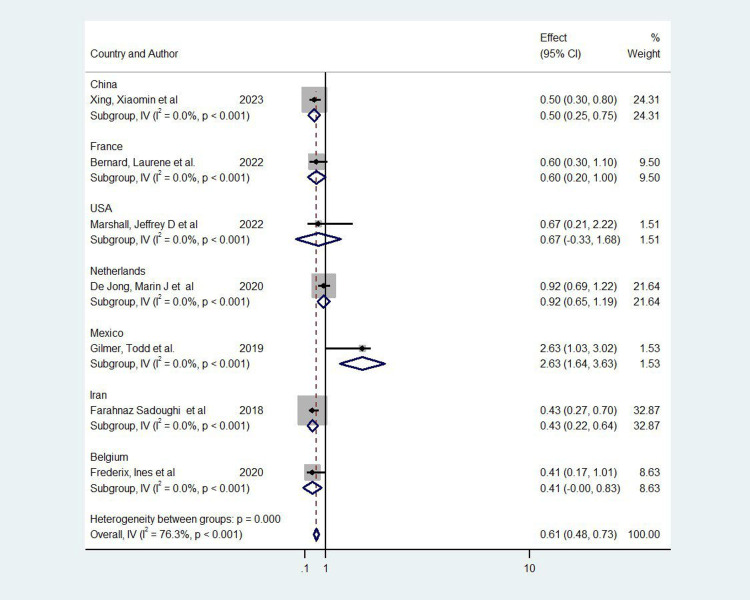
Forest plot of the included studies

In the meta-analysis, a total of seven studies were included [7-13], representing various interventions across different countries (Figure 2). The overall pooled odds ratio (OR) was 0.61 (95% CI: 0.48-0.73), indicating a significant positive effect of the interventions on the targeted outcomes. However, there was substantial heterogeneity between studies (I² = 76.3%, p < 0.001), suggesting variability in the intervention effects across different contexts. Notably, a study from Iran by Sadoughi et al. contributed the highest weight to the analysis at 32.87%, reflecting its larger sample size and higher precision, with an OR of 0.43 (95% CI: 0.22-0.64) [12]. In contrast, the study from the USA by Marshall et al. had a wider confidence interval crossing 1, indicating a less precise and nonsignificant effect (OR = 0.67, 95% CI: -0.33-1.68) [9]. Subgroup analyses by country revealed significant effects in studies from China (OR = 0.50, 95% CI: 0.25-0.75), the Netherlands (OR = 0.92, 95% CI: 0.65-1.19), and Mexico (OR = 2.63, 95% CI: 1.64-3.63), whereas the study from Belgium had a marginally significant effect (OR = 0.415, 95% CI: -0.004-0.834) [7,10,11]. These results demonstrate the overall effectiveness of the interventions but also highlight the variability in outcomes, potentially due to differences in healthcare settings and intervention designs across countries.

**Figure 3:**
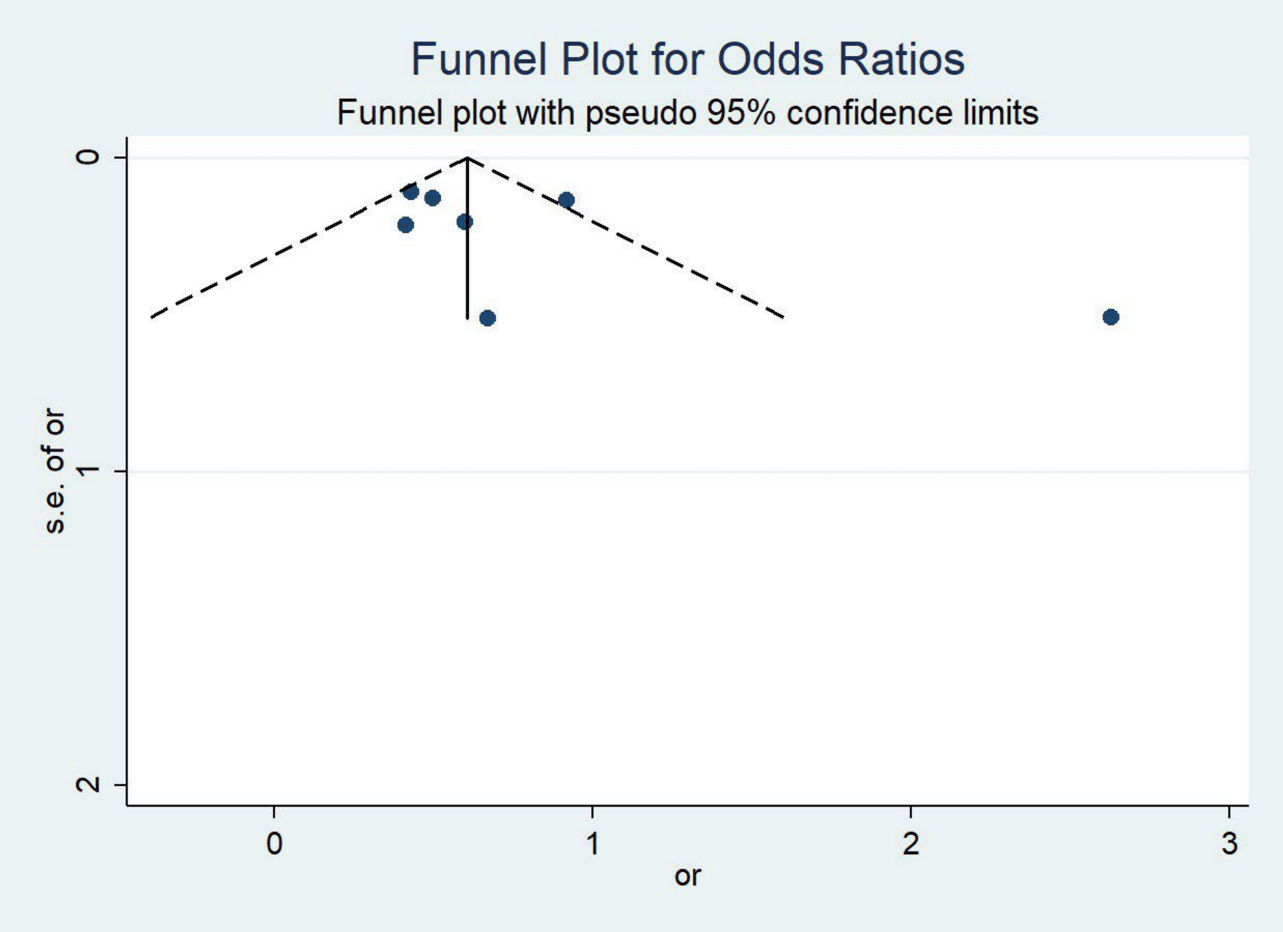
Funnel plot for Odds Ratios of the included studies

Figure 3 presents a funnel plot assessing potential publication bias in the included studies. The plot appears relatively symmetrical, with the studies distributed evenly around the pooled effect estimate. This suggests a low likelihood of publication bias. The majority of the studies lie within the pseudo 95% confidence limits, and no clear clustering on one side of the plot is observed, further supporting the robustness of the meta-analysis results. However, a few outliers can be seen at the right of the funnel, indicating the presence of larger effect sizes with higher standard errors in some studies. Despite this, the overall shape of the plot does not raise major concerns regarding bias.

Discussion

The findings of this study have significant implications for healthcare policy and practice. The evidence-based factors and strategies associated with supporting efficiency and service improvement, as outlined by Walters et al. [1], emphasize the importance of timely progress reporting, benchmarking, and transparent target setting. Furthermore, the study underscores the need for sufficient resourcing, data access, stakeholder engagement, and collaboration for successful change and system-wide improvement in efficiency. These insights are crucial pointers for policymakers and healthcare practitioners because they highlight the key areas that require attention and investment to enhance hospital efficiency and cost management.

Moreover, the systematic review by Campanella et al. [16] of the impact of public reporting (PR) on clinical outcomes provides valuable insights into the effectiveness of PR in hospitals and primary healthcare settings. Even though the studies that were evaluated were of poor quality, the review by Campanella et al. found that PR can have a positive impact on clinical outcomes in hospital settings and at primary healthcare level. This finding suggests the potential of PR to drive improvement in healthcare quality and outcomes. However, further research is needed to fully understand its mechanisms and effects in out-of-hospital settings. These findings can inform healthcare policies aimed at leveraging PR to enhance transparency and QoC delivery.

Staffing and workforce optimization are crucial aspects of hospital management, given that they directly impact resource allocation and overall efficiency. The literature offers several strategies for improving performance through human resource management practices. For instance, onsite team training in obstetric emergency care has been found to enhance team performance and clinical practices, leading to improved maternal outcomes [17]. In addition, the implementation of incentive systems and wage incentives for specialists has been linked to improved performance, more efficient service delivery, and greater staff retention. Furthermore, managing rostering and scheduling to control moonlighting has been associated with improved QoC and reduced intention to leave.

In the context of optimizing operating room performance, the challenges related to workforce management are evident. Healthcare professionals often face heavy workloads and limited autonomy. These two factors have a negative impact on their preparedness for significant changes in their work and contribute to high turnover rates [18]. The optimization of operating rooms is a key concern because of its impact on patient outcomes and the pressure on healthcare professionals. Metrics for assessing performance optimization range from efficiency indicators, such as the number of operations and total utilization time, to quality aspects that emphasize patient safety and high-quality care. Strategies such as precise scheduling and limiting resource waste have been highlighted, reflecting the importance of effective workforce management and resource utilization in hospital settings.

Technological integration and automation play a crucial role in enhancing hospital efficiency and operational processes. In other words, technology has the potential to reduce the nursing workload, improve care processes, enhance patient safety, and prevent staff burnout [19]. The integration of innovative technologies in healthcare systems disrupts traditional processes and management, ultimately leading to improved efficiency. In addition, researchers have recommended the promotion of smart healthcare, the utilization of artificial intelligence, and the implementation of big data analysis to optimize the use of healthcare manpower and resources, thereby reducing wastage and improving overall efficiency [20].

Cost management in hospital settings involves various approaches to optimize resources and streamline financial processes. Supply chain optimization is one crucial aspect, as highlighted in the Sant Camil Hospital case study [21]. This approach emphasizes the organization of hospital processes to enhance efficiency and customer satisfaction, drawing from concepts like Just in Time and Total Quality Control to minimize waste and enhance quality. Furthermore, budgeting and financial planning play a pivotal role in cost management, as evidenced by the findings of Homauni et al. [22]. These approaches collectively contribute to a comprehensive understanding of cost-management considerations within hospital settings, with the enhancement of operational efficiency and financial sustainability as the ultimate aim. However, Homauni et al.' study highlights challenges in capital budgeting techniques, such as prioritized repayment, ignoring total return on investment, and time value of money.

Sabwami et al. [23] also stress the importance of budgetary processes in resource allocation, performance measurement, and strategic management. On the one hand, Sabwami et al. emphasized the positive impact on hospital performance when strategic management and budgeting are integrated. On the other hand, the Sant Camil Hospital case study demonstrated the effectiveness of process management, which increased operating room occupancy by an average of 12% across specialties, thus reducing surgical waitlists [21].

Information systems play a significant role in facilitating the approaches discussed above, highlighting the importance of technological solutions in supply chain optimization within hospitals. Similarly, Rsuuo emphasizes the relevance of logistics management in hospital operations. The researcher stressed the need for efficient physical patient pathways and the transfer of logistical practices from manufacturing and service industries to the healthcare sector to improve the management of healthcare goods and patients [24]. These insights underscore the crucial role of supply chain optimization in enhancing hospital efficiency and cost management.

It is important to note that innovative cost management strategies not only impact the quality of services but also have the potential to considerably reduce financial wastage, particularly in categories such as overtreatment and administrative complexity. Various such strategies have been identified, but building synergies, standardizing processes to reduce waste, enhancing referral techniques, and reducing the length of stay have emerged as crucial steps to sustain quality improvement and cost reduction in healthcare organizations [25].

Furthermore, Walters et al. emphasize the importance of stakeholder engagement, addressing staff resistance, and collaboration in efficiency improvement processes. The researchers note that involving stakeholders in target-setting and initiative development, and prioritizing principles such as collaboration and performance monitoring, are associated with project impact and sustainable improvements in public health systems [1].

The systematic review by Mbau et al. highlighted the need for future research to include meta-analyses of interventions targeting health system efficiency. This inclusion would prevent potentially key literature from being overlooked [26]. Moreover, the literature review by Ali and Kannan emphasized the exponential growth of research in healthcare operations and supply chain management, indicating a growing interest in efficiency improvement and technology implementation within the healthcare sector [27].

These strategies offer valuable insights for healthcare managers seeking to improve efficiency and reduce costs within hospital settings, ultimately contributing to the overall organizational performance.

The major limitation within our study was the availability of a large number of relevant studies. Also, some studies within the review don’t report some valuable information, which can be very useful in improving hospital efficiency and cost management.

## Conclusions

The findings of this systematic review and meta-analysis offer valuable insights into the need for system-wide efficiency-improvement activities to be led by central management entities. In addition, the focus should be on service integration, collaboration, locally tailored solutions, and knowledge sharing between hospitals and internal departments. This study also highlights the significance of shared risks, goals, responsibilities, and feedback across public health systems to promote sustainable outcomes. The findings provide a foundation for policymakers to understand the evidence base that supports a systems approach to healthcare improvement. These insights can provide valuable guidance to hospital administrators and policymakers who seek to enhance efficiency and cost management within healthcare systems.
